# Health promotion in primary care: How should we intervene? A qualitative study involving both physicians and patients

**DOI:** 10.1186/1472-6963-11-62

**Published:** 2011-03-23

**Authors:** Carlos Calderón, Laura Balagué, Josep M Cortada, Álvaro Sánchez

**Affiliations:** 1 Centro de Salud de Alza, Comarca Ekialde, Servicio Vasco de Salud-Osakidetza, San Sebastián, Spain; 2 Centro de Salud de Iztieta, Comarca Ekialde, Servicio Vasco de Salud-Osakidetza, Rentería, Spain; 3 Unidad de Investigación de Atención Primaria, Servicio Vasco de Salud-Osakidetza, Bilbao, Spain

## Abstract

**Background:**

The effects of tobacco, physical exercise, diet, and alcohol consumption on morbidity and mortality underline the importance of health promotion and prevention (HPP) at the primary health care (PHC) level. Likewise, the deficiencies when putting such policies into practice and assessing their effectiveness are also widely recognised. The objectives of this research were: a) to gain an in-depth understanding of general practitioners' (GPs) and patients' perceptions about HPP in PHC, and b) to define the areas that could be improved in future interventions.

**Methods:**

Qualitative methodology focussed on the field of health services research. Information was generated on the basis of two GP-based and two patient-based discussion groups, all of which had previously participated in two interventions concerning healthy lifestyle promotion (tobacco and physical exercise). Transcripts and field notes were analysed on the basis of a sociological discourse-analysis model. The results were validated by triangulation between researchers.

**Results:**

GPs and patients' discourses about HPP in PHC were different in priorities and contents. An overall explanatory framework was designed to gain a better understanding of the meaning of GP-patient interactions related to HPP, and to show the main trends that emerged from their discourses. GPs linked their perceptions of HPP to their working conditions and experience in health services. The dimensions in this case involved the orientation of interventions, the goal of actions, and the evaluation of results. For patients, habits were mainly related to ways of life particularly influenced by close contexts. Health conceptions, their role as individuals, and the orientation of their demands were the most important dimensions in patients' sphere.

**Conclusions:**

HPP activities in PHC need to be understood and assessed in the context of their interaction with the conditioning trends in health services and patients' social micro-contexts. On the basis of the explanatory framework, three development lines are proposed: the incorporation of new methodological approaches according to the complexity of HPP in PHC; the openness of habit change policies beyond the medical services; and the effective commitments in the medium to long term by the health services themselves at the policy management level.

## Background

The effects of tobacco, physical exercise, diet and alcohol on morbidity and mortality in industrialised countries, and the subsequent resource investment, largely explain the growing attention being paid by health systems to the promotion of healthy habits and the assessment of their effects [1,2]. In the case of the Basque population older than 16 years of age, the 2007 Health Survey found that 53% were sedentary in their free time, 49.6% were overweight, 25% smoked regularly and 8.2% consumed alcoholic beverages in large or excessive amounts [3]. These unhealthy behaviours vary according to sex and social class and frequently co-occur in the same individual [4].

The characteristics of primary health care (PHC) concerning treatment accessibility and continuity of care, place it at an ideal position to potentially intervene to improve these habits [5]. In Spain, the Spanish Society for Family and Community Medicine (semFYC) established the Health Promotion and Prevention Activities Programme (PAPPS) in 1988 to be applied in health centres and by PHC professionals [6]. Since then, the health services have incorporated various computer programmes to monitor its implementation.

However, the reality of putting into practice and assessing the effectiveness of health promotion and prevention (HPP) activities at the PHC level is not trouble-free. Their incorporation into the daily schedule of GPs is limited, with lack of time and excessive workload as causes mentioned most often [7]. Assessments of their effectiveness in different contexts have, in general, provided inconclusive results [8,9].

As a result, it appears necessary to design new intervention and assessment models that are better able to take into account the characteristics of the setting in which they are to be undertaken [10]. Specifically, as regards the role of GPs, several qualitative studies have highlighted the secondary and rather unsatisfactory role played by HPP activities [11,12], some doubts regarding their effectiveness based on factors unrelated to healthcare [13], and their possible interference in the physician-patient relationship [11,14]. Concerning patients' perceptions, the importance of the social and cultural context and the existence of communication barriers between patients and physicians have been brought to light [15].

To the best of our knowledge, no previous studies have involved both patients and physicians simultaneously in the same context. Developing an understanding of their experiences under these circumstances will therefore allow us to define a framework to explain the discourses of both these agents and identify the main factors that should be considered in the future implementation of HPP at a PHC level.

This qualitative study corresponds to the second phase of the commissioned research project "Useful strategies for promoting healthy lifestyles in PHC" [16], undertaken by the Primary Care Research Unit of the Basque Health Service/Osakidetza (UIAP). The first phase of the project was already published [17]. Although both studies were performed simultaneously, the specific objectives of the present research were: a) to gain an in-depth understanding of the perceptions of GPs and patients as regards HPP in PHC, and b) to define the main areas that could be improved in future interventions.

## Methods

The relevance of qualitative methods to the field of HPP has been previously highlighted [18]. In this case, the qualitative methodology was focused on the field of health services research [19] and PHC [20], and discussion groups were considered to be the most appropriate technique for generating information. Discussion groups are a group-based technique similar to the focal-groups but aimed more towards encouraging the interaction and interpretation of the different positions held by the participants [21].

This study involved GPs and patients who had previously participated in two habit change interventions undertaken in PHC in Biscay (Basque Country): the assessment of the effectiveness of the Stop Smoking Programme (PAT) undertaken in 1996 [22], and the Experimental Physical Activity Promotion Programme (PEPAF) undertaken in 2003 and 2004 [23].

Two groups of GPs and two groups of patients were formed in order to contrast the findings and assess the degree of saturation reached. After explaining the study goals and background, 5 GPs from the PAT study (discussion group D1) and 8 from the PEPAF study (D2) agreed to participate. They were 6 women and 7 men, their ages ranged from 47 to 53, and the time worked in PHC from 15 to 27 years.

Patients were selected using a stratified sampling method on the basis of their GP, age group, sex and change or not of the habit in question. The selected patients and their GPs received a letter explaining the aims of the study and requesting their participation. No ethics committee approval was required in this study as it was a service evaluation commissioned by the Health Department of the Basque Government. Prior to giving their consent, all patients were informed of the ethical requirement that participation in the study would not affect their care and that their confidentiality would be guaranteed.

Finally, two patient groups, one (P1) consisting of 9 patients who had changed their habits (5 from PAT and 4 from PEPAF) and the other (P2) 6 who had not (3 each from PAT and PEPAF), were formed. Patients were 7 women and 8 men, their age ranged from 45 to 80, their educational levels were Primary (4), Secondary (6) and Degree (5), and regarding activity 7 were active, 4 retired and 4 housewives. The existence of different levels of education did not interfere with the interaction and participation within groups.

All groups met in October and November 2006 at the headquarters of the UIAP in Bilbao. Sessions lasted for around 90 minutes and were attended by two trained researchers, one of whom acted as moderator and the other as observer. After the initial presentation, basic sociodemographic data were collected, stressing the confidential nature of this process, and permission sought to record the session. The discussion groups were based around a flexible guide open to new topics and they finished once the participants considered that they had nothing further to add. After each meeting, the researchers made brief notes about provisional findings and group dynamics.

The analysis was performed on the basis of the transcripts and these notes using a sociological discourse-analysis model [24,25]. The interpretative work in this model began with an overall view of the texts and their relationship to the context in which they were produced. A repeated and detailed reading of the transcripts allowed the different discursive positions to be identified and coded, and the relationships, corroborations and contradictions between them to be determined. Cognitive maps [26] were subsequently designed in order to build explanatory schemes, initially for each group, then for each sub-population (physicians and patients) and finally for all participants together. Each step in this abstraction-interpretation process was contrasted iteratively with the empirical material as a whole in order to detect possible negative cases and to determine the degree of saturation of each category. Triangulation between the researchers who participated in the data collection and analysis process was used as a complementary validation procedure [27,28]. Full transcripts can be found in the report published by the Department of Health of the Basque Government [16]. It can be seen from this report that the role of other groups, such as nurses, was also considered during these discussion groups, although here we concentrate solely on GPs.

## Results

GPs' and patients' discourses about HPP in PHC differed in priorities and contents. We therefore arranged the main findings of analysis in two sections. The first one was centred on GPs' perceptions and the second one on those of patients. In both cases HPP was the focus, but it was also important to know their mutual discourses about the role of patients and GPs in PHC. GPs linked their perceptions of HPP to their working conditions and experience in the health services. From their perspective, patients' attitudes and contexts added difficulty to a very often frustrating duty. For patients, habits were related to ways of life particularly influenced by close contexts. GPs' role was regarded as important but secondary. GPs had to be experts in helping them when necessary, but their own demands and responsibilities were not homogeneous.

### 1. Physicians' perspective: between duty and frustration

GPs' discourses were located in the context of health services and PHC centres. Their experiences about these services and the patients who, in many cases, they had treated for many years were particularly relevant. Contradictions between what should be done and what was actually done appeared frequently with a notable sense of frustration related to the current situation.

#### a) Health promotion and day-to-day working conditions

GPs were generally aware of the importance of HPP and the closeness and continuity of their handling of patients. However, the very nature of their care work made it difficult to incorporate HPP activities. GPs had first of all to respond to the demands considered as a priority by patients, which often meant that HPP was relegated to second place. In this sense, lack of time frequently appeared as a problem related to the characteristics of PHC and to the current HPP intervention and assessment models.

"-...To be honest I think that we see people a lot, on numerous occasions, and it is then when we can get involved and maybe identify the patient at risk... The motivation is often external. and that's when we can get involved...I believe that our advantage is that we are always there..." F2D2

"-...a patient isn't a single consultation, a patient can be seven consultations,... that back pain you have just there.... because if you take the back pain seriously, you then sort out the other one and the other one, so it´s not 10 minutes... So we have a mixture of things. In prevention, personally I have my four little elements." M2D1

"-...Well, the time... I have another problem... I find it difficult to perform preventative activities, not because I don't believe in them... it's just that I can't, I forget.... I remember that I have to do the PAPPS just as the patient is leaving...it's difficult... For me the consultation is something that I really get into, the reason for that day's consultation, I get caught up in the topic and when it's over the consultation has finished, it's difficult for me: "and am I going to ask the patient now?" ... My graphs aren't very good..." M1D2

GPs' accounts of their participation in the two previous studies made also frequent mention of the lack of time and additional workload they faced, with the subsequent doubts regarding their long-term feasibility. However, at the same time their impressions regarding their design, learning component and novelty with respect to the routine were favourable.

-"...The work was difficult for me personally at the time as it meant an increased workload... Everyone tells you when you participate in something like this that it's very easy. Well it's not." H2D2

-"... We learned everything we know there...But we must have ended up exhausted, because when the two years were up, because it lasted two years, there was an enormous shift wasn't there? The following year I think I was a bit scared to ask "would you like us to help you?"...Jesus, if they say yes, then tomorrow at 8:30, from 8:30 until 9:00 I'll be busy..." M2D1

-"...It seemed a good idea..., furthermore smoking was an important topic for me...for various reasons. I was already a bit fed up at that point with the protocols...and this represented something different...later I learned a lot..." M1D1

In other words, doubts regarding the conditions in which HPP was undertaken concerned both general resource-demand aspects and the management of these activities. The concept of HPP as an "ideological" imperative was questioned on the basis of the effectiveness of its actions, particularly when these actions were compared with the area of disease treatment.

"-...I think we shouldn't concentrate so much on success rates...at least in the short term... it's something we have to do because we believe in it, that's all... There must be some sort of ideology behind this...M1

-But we have to know whether things work or not! That's dangerous as well...I mean, maybe we're achieving something... and there should be some way of measuring it...." M1D1

"...What is the goal in health promotion?...That's what's worst, because we know what the goal is with pneumonia, we know what figure we need to reach with high blood pressure and so on...but for smoking... how many people are supposed to stop smoking with our help?...or how many are going to stop drinking? I don't touch alcohol any more, it terrifies me, I don't know about the rest of you..." F3D2

HPP activities assessment was a clear reason of discontent, and the design of the computer system used for this purpose in PHC was also questioned. In some cases this system was recognised useful as a reminder, but over all it was seen as unhelpful and even leading to the "devaluation" of HPP itself as data entry was considered as an end, rather than a means.

"-...The computer system... is going to end up like this, as form-filling. I don't see it as being particularly useful... Maybe if the computer system wasn't there you wouldn't even weigh [the patient]...but that's all...I don't believe the main point of medicine is to tick boxes... M2D1

"-...We've got a computer system containing all the preventative activities...that's a disaster....F3D2

-...And only what you enter is assessed.......and you can only enter what it lets you enter...the rest therefore doesn't exist...M1D2

-...We have to survive...M3D2

-...But if it doesn't matter why bother asking....as long as you tick the box...M1D2

-...So, what's important is to tick boxes?...F1D2

The duty imperative and frustration were located in this case in the requirement to fill in records that did not accurately reflect the role played by GPs in HPP activities.

#### b) Habits, patients, and their contexts

From GPs' perspective, patients may present various unhealthy habits whose management was not easy at all in light of the variability of their experiences, contexts and motivation to change. HPP activities were therefore perceived as "swimming against the tide", highlighting the importance of personalised treatment, empathy and opportunity concerning how and when to intervene.

**"-**...any change of habit requires facing up to a similar type of problem... overweight, obesity, a sedentary lifestyle, diabetes, pre-diabetes, a whole range of things... But all these possibilities suppose a lifestyle change.... so it feels a bit like always swimming against the current... F1D1

"-...And in the end, the culture and society in which we live, and the empathy you have with your patients in general, come into play....in other words, as we were saying earlier, if you connect with a patient you have more chances of changing that patient's habits...if you get on well with someone they take more notice of you...M1D1

"-...I think we know a lot about how to do things but not much about the background... I mean, I get stressed when a patient comes to me because he's losing weight and has a lymph node problem,...and then you go and ask him if he drinks...in other words,...I don't know... other things worry me more than the macro data..."M2D2

References to the social context in GPs' discourses located the problem beyond the scope of consultation. The importance of their effects on individual behaviour was reflected in the expectations and arguments with respect to what can be achieved at the PHC level. Frustration also emerged in GPs' accounts who, on the one hand, understood HPP as part of their day-to-day duties, and on the other, felt that a large part of the responsibility for the final success or failure lied outside their scope of action.

"-...We may want to undertake promotion and prevention, but we have to realise that it is extremely frustrating to find that, when the individual habit is indeed a habit, it's very difficult to change....In this case we still have to try, but health education must also be much more society oriented,... it must come from much higher than primary care... F2D4

"-... it's complicated, ok?. In other words, the epidemiological mechanisms...they tell you that the real power of preventative medicine isn't in the physicians' hands but in those of the Minister of Public Works and people like that....and that intervention at a population level is better than at an individual level. And as physicians we have to accept that, because if not, we get extremely frustrated....M3D1

Tensions between the "inside" and "outside" PHC poles made GPs ask themselves about how the effectiveness of HPP was assessed. References to resources external to health services and the convenience of coordinating efforts were also present in their discourses.

"-... I believe that we have indeed managed to convince patients that physical activity is important...as I now find... some patients... who you'd never see out walking previously and who now, with their sixty something or more years, go out for a walk...What percentage? I've no idea. Was it because of this? Probably not entirely... M3

- Can you now find elderly women in tracksuits because of this research? Surely it's for other reasons, isn't it? M2

-...Certainly. But to what degree? Who knows...M3D2"

"-...I think that, without trying to avoid the matter at hand, that there's something else that everyone's aware of, and that's community-based intervention.... I believe that town halls and the like should intervene as these are health habits...and, if they decide to do that, parallel activities aimed at the community which don't just involve ourselves...It would be the same as the positive effects we've seen with the anti-smoking laws...M2D1

However, their perceptions about the future were sceptical. Interventions at the community level were considered, in some cases, as utopian, whereas in others emphasis was placed on the need of support resources.

-...I think we would be very good there, like some sort of linchpin between disease and health and such... to bring together health resources and the population, that's nice work....the only problem is that that's an utopia....M2D2

-...But it's complicated, you can't of course do it now...M1D2

-...Well, it all depends on interests from above, obviously, and the resources they give us, but in the state we are at the moment, nobody in their right mind would organise something like this...M3D2

-...It's difficult to try and fit this in to the schedule because it seems to me that as you get older you're less willing to volunteer your time... I mean, it's not the same you having to give a talk at 7 o'clock as your manager telling you "I'll give you 3 hours off and you can give the talk when you can"... You need time, money...because then you get the acute [cases], the colds, and you can't ignore them....M1D1

### 2) Patients' perspectives: feeling healthy and fear of disease in complex contexts

Patients' perspectives of HPP were more open and plural. Their role as individuals linked to specific socio-cultural contexts was clear in both groups. Their accounts often referred to habit changes on time scales different to those of interventions, and the reasons that led them to change differed in terms of priority and opportunity from those of GPs. The concepts of promotion and prevention were replaced by experiences with blurred boundaries between feeling healthy and the fear of disease.

#### a) The importance of micro-social factors

The strong influence of the social context corroborated GPs' perceptions, although for patients these factors seemed to be more complex and closer to micro-contexts. References to known cases about relations between habits and the likelihood of suffering a disease were especially visible.

"-...I was already being monitored, and on top of that the doctor encouraged me to do exercise..., but the cholesterol was something that I was already aware of... Two workmates of mine died from heart attacks and the last one terrified me, so I stopped smoking around two years ago. M4P1"

"-...it's not an excuse but... my father smoked all his life and lived to 85, and my father-in-law is still smoking at 91... so I haven't really seen anyone die young, therefore there's been no immediate reason to stop smoking right away." M2P2

This area included factors related to local customs, the socio-economic level, family, work, friends and gender roles which complicated the motivational map and the possible causal factors.

"-...We, at least our generation,... have this culture of eating, and when we get bored and don't know what to do we eat and drink, and when we're not bored because we're going out on the town, we eat and drink... M1P1

- I don't think so, each to his own...As a housewife my case is completely different, I have very little time to go out because I can't, I've got things to do, but my problem is anxiety... I'm either stressed because things aren't working out as expected or because of family problems... F2P1

- I work as a cleaner and when I get home it's more of the same. It's a routine which might just take you along with it... F3P1"

"-... I stopped smoking in solidarity with my husband and children..."F5P1

Clear attitude differences were seen as well in terms of a greater/lesser assumption of responsibility for their habits. In some cases decisions were accepted to be their own, whereas in others it was easier to project this responsibility externally.

"-...I smoke a lot, at least a packet, sometimes I overdo it...At times I tell myself "I'm going to stop smoking" and then I go and see the doctor... but then I say 'but if I don't really want to stop, what's the point of wasting the doctor's time if I'm really not motivated?" F2P2

"-... I think they should have provided help, or a treatment or support groups for those of us with a smoking problem to convince us to stop smoking rather than just coming up with a law,... F4P1"

The influence of external factors was occasionally located at a macro level, especially for smoking. Perceptions of feeling stigmatised were used, in some cases, as a reaffirmation, highlighting what they considered as "inconsistencies" with respect to other health problems. In others, their right to make decisions concerning their own habits was also argued in their "defence".

*"-...I understand that it's difficult for someone to come up to me and call me an alcoholic because of the social rejection that goes with it. A smoker doesn't have that problem M3P1*.

-...Somebody once complained that I was smoking in the street...in the street!." F4P1

"-...This goes in cycles, they bother you, it's a cycle... and suddenly you've got a cold because you smoke, you've got whatever because you smoke... everything is because you smoke. M1P2"

*"-... when they keep insisting on how bad smoking is for you, you sometimes want to blow smoke in their face...... taking drugs is also bad for you, and roast suckling pig is terrible, and if you go to Segovia and don't eat it you're an outcast... Let's be a bit more logical here! Let's point the finger at everyone, not just at me...M2P2*.

-...You're not going to tell me that now they're worried about us, they've never worried before... why bother worrying about smokers. For God's sake, worry about people who are dying of hunger, 4000 km from here they're dying of hunger, worry about them..." M4P2

The influence of diseases or disabilities related to unhealthy habits emerged as a clear discursive motivation for change, although the parameters related to "being healthy" were not as simple as "yes/no" or "good/bad" dichotomies.

*"-...I smoked for maybe 15 years or more until one day I said, this can't go on, this looks like it's leading to cancer so I have to stop smoking... I went to buy my packet of cigarettes with bronchitis, on the point of developing cancer...and I simply put the packet in my handbag... One day I went to the doctor's and he said to me 'So, have you stopped smoking yet?' 'Yes, I've given up, I haven't smoked for almost a year"F3P1*.

"-...I also play sports and so obviously... when my doctor told me about stopping smoking I had no intention of stopping, just like now... Everyone needs a vice, it's not as if I eat or drink too much... I often go into the high mountains and I don't feel unwell... I get on ok... but I walk a lot better than many people who don't smoke,..." M4P2

"-...I don't smoke a lot, six or seven a day at the most and I've never been pressured into stopping... although I'd never defend smoking. I had a spirometry test just over a year ago and I was at 100%, I had just over 98% lung capacity, although that's not to say I'm defending anything, just that I haven't noticed anything..." M2P2

#### b) The GPs' role

Even when the influence of social environment was overwhelming, especially at the micro-social level, the health care was recognised as an important reference concerning habit maintenance or change. Patients viewed their GP's work in the HPP field favourably; indeed, GP's influence when deciding to participate in the two previous studies was clear. However, awareness of its limited effects also appeared in their discourses.

"-...Basically because my GP asked me to participate, not for any other reason. I went to see my GP and he asked me 'How would you like to participate in some projects?', 'Well, ok then'. As I had time, I went. F1P1"

"-...That's the GP's role, to help you, to be the one who's always insisting, and who says to you every time you visit '...What's my GP going to do, to force me? No... then to be so insistent that in the end I ask what he wants. That's his role....M1P1

- No, I don't think the GP has much of an influence, it's us who have to....it doesn't matter how much he insists.... he can help a bit but that's all. F1P1

- I think he does help because it's him who tells you what to do, although whether you then take any notice of what he's telling you, or whether you're able to do it...F4P1

- The only thing the GP can do is advise you, and advise you well, but everything else has to come from you...or give you a bit of a fright...M2P1"

GP's advice was therefore somewhat expected, and to some extent welcome, but it was treated as an advice that can be followed or not and, furthermore, which loses its effect if given indiscriminately without considering each patient's circumstances and characteristics.

"- I once knew a GP who said to an 85-year-old who smoked four cigarettes a day - it was his only vice and he was practically at death's door anyway: 'Do you smoke?' 'Yes, four [a day]' 'Well you should stop'. With all due respect, you feel like saying to the GP 'stop working'... it doesn't seem very logical to me. M1P1"

"-... I think that for a person to give advice about something, that person should have some degree of moral authority to do it. I mean, if you go to a doctor it's because he is able to help you on health matters. So, if he says to you clearly 'you shouldn't smoke'...But it shouldn't be that every time you visit it's like...'my back hurts', 'ok, but you shouldn't smoke' (laughs) M2P2"

The fear of disease was also present in their communications with health professionals, and not only with their GPs. This focus on disease was also present in the reasons why some patients decided to participate in the previous studies, regarding the possibility of complementary tests (analyses, etc.) as a better prevention.

"-...my GP, who I've known all my life, has been continually trying to get me to stop smoking... smoking didn't affect my physical activity... until one day I had a sore throat,.... I went to see my GP, who sent me to see a specialist who scared the life out of me. The specialist told me he was going to make a small hole, and since that day, July 16th at half past nine in the morning, I haven't smoked a single cigarette." M1P1

"-...I've had a snoring problem for a long time... Obviously, my GP said I should stop smoking... In the end, I went to the ENT specialist and finally needed an operation on my vocal cords... But I carried on smoking... Then, one day, he told me about the small hole, you're going to end up with a small hole. I don't know whether it was because it scared me so much or because he got me at a good moment, I don't think I'll ever know to be honest, but I decided to stop smoking on a certain date. That was a year ago" F2P1

"-...My GP encouraged me to participate in this programme. I was having my cholesterol monitored, it's a bit too high. He also encouraged me to take more exercise and he told me that every time I attended this programme they would do another control... When he told me that I thought, great, because as well as coming here to see how I'm getting on with my cholesterol, then this programme will monitor other things... One year is a long time..."M4P1

## Discussion

The information analysed above was generated in a context, and circumstances, that should not be overlooked when assessing their applicability. However, the findings highlight some aspects whose interest transcends the health centres where participants were recruited. The meanings identified, and their relationships, allow us to define an overall explanatory framework and to discuss possible lines of action which should be considered in the future.

### Characteristics of the explanatory framework

**Figure 1:**
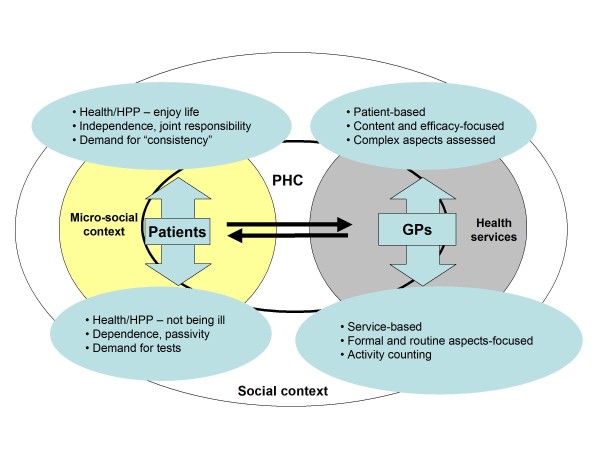
**Relationships and trends in healthy lifestyle promotion**.

Figure 1 shows the main dimensions and "meaning axes" that affect HPP in PHC from the analysis of GPs and patients' discourses.

Within the proposed framework, HPP activities form part of the "horizontal" relationships that GPs and patients develop in the area delimited by PHC, although the explanation of their practices and behaviours exceeds these limits in both cases.

In the case of GPs, HPP activities are always defined by their working conditions and current policies in the *health services*. References to work overload and lack of time noted in other studies[11,29] are also present here. Demand is plural and linked mainly to illness, therefore GPs tend to "get hung up on" their resolution, relegating HPP to a more secondary role [30]. Doubts about the effectiveness of standardized HPP activities contribute to add frustration to duty. However, both, reasons and consequences, can be better understood on the basis of the "vertical" trends shown schematically in Figure 1.

As HPP activities tend to be more patient-based, they are perceived to be more effective, although this approach is more difficult and time-consuming than routine work. Personalisation of HPP activities, taking into account the characteristics of each patient and their social and family context, acquires particular importance. Furthermore, a long-term relationship between GP and patient generates an environment of mutual expectation and complicity conditioning the effects of interventions depending on who, how and when they are undertaken [29].

Difficulties stemming from individualization are not reflected in the programmes and evaluations based on counting the number of activities, as long as they are oriented more towards the formal requirements of health services than those of patients themselves[31]. The lack of correspondence expressed by GPs between "the graphs" and actual nature of HPP work, and the uncertainty regarding the effectiveness of activities distanced from the complexity of involved factors [11,12,32] are also located in the "vertical" axis of GPs' discourses.

Discourses of patients are mainly generated in the *micro-social *area, with a marked influence of family and work contexts, friend networks and hobbies. Factors such as gender and socio-economic level, age profile (medium-high) and urban environment in which they live, with no major difficulties concerning access and services, condition their arguments and narratives. The role of the husband/wife and children, as well as leisure habits, strongly linked to friendships and to the social role of food and drink, should also be taken into account. An understanding of the main "vertical" trends in this so-called micro-social context (Figure 1) is therefore necessary.

Thus, as the discourses become more oriented towards identifying health with well-being, enjoyment and pleasure, disease prevention-based medical advice tends to be more challenged. In some cases, examples were sought to contradict this advice or to question its indiscriminate content. This demand for "consistency" is aimed more towards highlighting other lifestyle- and work-related health problems that do not receive the same amount of attention.

However, tendencies to identify HPP with the avoidance of serious diseases were also found in patients' narratives. Indeed, on occasions it is the main reason for changing their habits. This link between HPP and "not being ill" also explains its projection to the demand for more tests and check-ups[33], as well as the attraction that the participation in studies which involve a greater number of controls may have in these cases.

Another trend present in the patients' discourses axis is the greater or lesser degree of self-responsibility concerning HPP. As we saw above, patients were generally aware of their own role in maintaining and changing habits[15]. Demands about their right to make decisions concerning their own lifestyle, with the subsequent rejection of outside interference, were even seen from some discourses. But in this dimension we also saw contrasting discourses, where a greater passivity and dependence on external factors caused the projection onto GPs and health services of responsibilities that do not correspond to them.

According to the proposed framework, the discourses reflecting GPs' and patients' perceptions about HPP in PCH are neither linear nor dychotomic. The "horizontal" relations in PHC interact with "vertical" trends generated in health services and patients' micro-contexts. As a consequence, different discourses can be present simultaneously in the same person and that makes it even more important to individualize the implementation of HPP strategies in PHC.

### The need to rethink new intervention and assessment programmes

On the basis of this shared trends framework it is possible to identify certain elements to be taken into account when designing future PHC-based HPP activities.

First of all, *new methodologies *better suited to the complex nature of HPP should be developed [29]. This complexity has to be considered in the design and assessment of change interventions [10,31,34] as well as in the theories to understand them [35-38]. For example, the use of the evidence-based scores from clinical trials, which were better known and expected by some of the GPs, may not always be the most appropriate way for evaluating HPP activities in PHC [39-41]. Also, some components of well known theoretical models as the health belief model, the socio-ecological model, or the social learning model [16], may have been identified in patients' discourses, but no isolated model can explain their meaning by themselves as all of them appeared intermingled.

The need to take complex factors into account undoubtedly adds difficulties to the research and assessment models used in HPP activities. However, it also makes them particularly necessary in order to overcome their current secondary role in PHC and to avoid the unexpected effects of the measures that are sometimes adopted by health services[31,34,42].

Secondly, some *areas beyond the medical services *should also be considered during both the theoretical debate and the future practical development of HPP in PHC[10,43]. The importance attributed to factors outside health services in the discourses of GPs and patients should be taken into account in the design of HPP interventions. This would result in a greater "consistency" and effectiveness [44] and would also help to articulate and drive new models of participation and joint responsibility for GPs and patients[45]. The challenges are not insignificant due to both the scepticism from professionals used to working under conditions that do not consider such aspects, and the requirement of the coordination and commitment of various bodies and institutions to put them into practice.

Finally, the *health services *themselves should develop HPP proposals which take into account both complexity and rigour when designing activities to be undertaken by GPs at the PHC level and which should be open to the coordination and participation of agents beyond the health sector. The ambivalence found in the GPs' discourses as regards their participation in the two previous studies reflects the importance of well-reasoned proposals, monitoring their progress[46], and reassessing working conditions to be undertaken effectively in the long term. In this sense, we insist on the insufficiency of considering only formal aspects or simply counting the activities done. It would also be appropriate to reinforce the cooperation and dialogue between PHC GPs and other healthcare (nursing staff, specialists, etc.) and non-healthcare professionals who become involved in interventions with the same patients and who therefore require coherence and collaboration[13,47].

## Conclusions

The discourses of both GPs and patients concerning habit changes and HPP policies have been used to construct an explanatory framework containing the dimensions and trends which intervene in PHC as a meeting point for both these agents.

In the case of GPs, these trends depend on the characteristics of the health service itself, and their bi-directionality is reflected in three main dimensions: the orientation of interventions (towards the characteristics of patients/towards the requirements of services), the goal of actions (effectiveness-based/concentrating on formal aspects), and the evaluation of results (incorporation of complexity/limited to activity counting).

The trends observed in patients' discourses are mainly linked to their micro-social context, and the main dimensions in this case are based on health conceptions (oriented towards well-being and enjoyment of life/based on avoiding illness), the patient's role when considering habit change (assuming independence and joint responsibility/showing passivity and a dependence on external factors), and the orientation of their demands (recognition of consistency/request for more health interventions).

Three lines of action which should be promoted in the future have been proposed on the basis of this explanatory framework. The first of these involves new methodologies for the development and evaluation of habit change policies that take into account the complexities inherent to the HPP field. Secondly, the importance of aspects beyond medical services, such as socio-cultural characteristics and the amount of resources available locally, should be considered alongside general legislative measures. Finally, the need to make effective compromises in health services at the policy management level to promote coherent mid- to long-term strategies aimed towards the patient and based on collaboration between the various agents involved should be highlighted.

## Competing interests

The authors declare that they have no competing interests.

## Authors' contributions

CC contributed to the study design, participated in data collection and prepared the preliminary version of the data analysis and interpretation. LB and AS helped to organise the various groups and collaborated in data collection and discussion of the results analysis. JC participated in the discussion of the analysis. All authors have read and agreed on the final version of this manuscript.

## Pre-publication history

The pre-publication history for this paper can be accessed here:

http://www.biomedcentral.com/1472-6963/11/62/prepub

## References

[B1] WHO The World Health Report. Reducing risks, promoting healthy life 2002 Geneva: World Health Organization

[B2] National Institute of Public Health and National Food Administration Background material to the action plan for healthy dietary habits and increased physical activity 2005 Uppsala. Sweden: National Food Administration

[B3] Departamento de Sanidad del Gobierno Vasco Encuesta de Salud de la Comunidad Autónoma del País Vasco 2007 2007 Vitoria-Gasteiz: Servicio Central de Publicaciones del Gobierno Vasco

[B4] Galan I Rodriguez-Artalejo F Diez-Ganan L Tobias A Zorrilla B Gandarillas A Clustering of behavioural risk factors and compliance with clinical preventive recommendations in Spain Prev Med 2006 42 343 347 10.1016/j.ypmed.2006.01.018 16545444

[B5] Starfield B Shi L Macinko J Contribution of primary care to health systems and health Milbank Q 2005 83 457 502 10.1111/j.1468-0009.2005.00409.x 16202000 PMC2690145

[B6] Sociedad Española de Medicina Familiar y Comunitaria PAPPS: Programa de Actividades Preventivas y de Promoción de la Salud 2009 http://www.papps.org/

[B7] Lopez-de-Munain J Torcal J Lopez V Garay J Prevention in routine general practice: activity patterns and potential promoting factors Prev Med 2001 32 13 22 10.1006/pmed.2000.0777 11162322

[B8] Ashenden R Silagy C Weller D A systematic review of the effectiveness of promoting lifestyle change in general practice Fam Pract 1997 14 160 176 10.1093/fampra/14.2.160 9137956

[B9] Goldstein MG Whitlock EP DePue J Multiple behavioral risk factor interventions in primary care. Summary of research evidence Am J Prev Med 2004 27 61 79 10.1016/j.amepre.2004.04.023 15275675

[B10] Jackson SF Perkins F Khandor E Cordwell L Hamann S Buasai S Integrated health promotion strategies: a contribution to tackling current and future health challenges Health Promot Int 2006 21 Suppl 1 75 83 10.1093/heapro/dal054 17307960

[B11] Williams SJ Calnan M Perspectives on prevention: the views of General Practitioners Sociol Health Illn 1994 16 372 393 10.1111/1467-9566.ep11348775

[B12] Lawlor DA Keen S Neal RD Can general practitioners influence the nation's health through a population approach to provision of lifestyle advice? Br J Gen Pract 2000 50 455 459 10962782 PMC1313722

[B13] Ribera AP McKenna J Riddoch C Physical activity promotion in general practices of Barcelona: a case study Health Educ Res 2006 21 538 548 10.1093/her/cyl008 16702195

[B14] Aira M Kauhanen J Larivaara P Rautio P Differences in brief interventions on excessive drinking and smoking by primary care physicians: qualitative study Prev Med 2004 38 473 478 10.1016/j.ypmed.2003.11.023 15020181

[B15] Brown I Thompson J Tod A Jones G Primary care support for tackling obesity: a qualitative study of the perceptions of obese patients Br J Gen Pract 2006 56 666 672 16953998 PMC1876632

[B16] Grandes G Sánchez A Cortada JM Calderón C Balagué L Millán E Estrategias útiles para la promoción de estilos de vida saludables en Atención Primaria de Salud. Investigación Comisionada 2008 Vitoria-Gasteiz: Departamento de Sanidad, Gobierno Vasco Informe no Osteba D-08-07

[B17] Grandes G Sánchez A Cortada JM Balagué L Calderón C Arrazola A Is integration of healthy lifestyle promotion into primary care feasible? Discussion and consensus sessions between clinicians and researchers BMC Health Services Research 2008 8 213 18854033 10.1186/1472-6963-8-213 PMC2577098

[B18] Gendron S Transformative alliance between qualitative and quantitative approaches in health promotion research WHO Reg Publ Eur Ser 2001 107 121 11729768

[B19] Murphy E Dingwall R Qualitative Methods and Health Policy Research 2003 New York: Aldine de Gruyter

[B20] Calderón C Fernández de Sanmamed MJ Martín Zurro A, Cano Pérez JF Investigación Cualitativa en Atención Primaria Atención Primaria. Conceptos, organización y práctica clínica 2008 Barcelona: Elsevier 211 240

[B21] Callejo J El grupo de discusión: introducción a una práctica de investigación 2001 Barcelona: Ariel

[B22] Grandes G Cortada JM Arrazola A An evidence-based programme for smoking cessation: effectiveness in routine general practice Br J Gen Pract 2000 50 803 807 11127170 PMC1313821

[B23] Grandes G Sanchez A Sanchez-Pinilla RO Torcal J Montoya I Lizarraga K Effectiveness of physical activity advice and prescription by physicians in routine primary care: a cluster randomized trial Arch Intern Med 2009 169 694 701 10.1001/archinternmed.2009.23 19364999

[B24] Alonso LE La mirada cualitativa en sociología. Una aproximación interpretativa 1998 Madrid: Editorial Fundamentos

[B25] Conde F Análisis sociológico del sistema de discursos 2009 Madrid: CIS

[B26] Walker R Applied Qualitative Research 1985 Hants: Gower

[B27] Morse JM Barrett M Mayan M Olson K Spiers J Verification strategies for establishing reliability and validity in qualitative research IJQM 2002 1 2 http://ejournals.library.ualberta.ca/index.php/IJQM/article/view/4603/3756

[B28] Calderón C Assessing the Quality of Qualitative Health Research: Criteria, Process and Writing Forum Qualitative Sozialforschung / Forum: Qualitative Social Research 2009 10 17 http://nbn-resolving.de/urn:nbn:de:0114-fqs0902178

[B29] Ampt AJ Amoroso C Harris MF McKenzie SH Rose VK Taggart JR Attitudes, norms and controls influencing lifestyle risk factor management in general practice BMC Fam Pract 2009 10 59 10.1186/1471-2296-10-59 19706198 PMC2746183

[B30] Jaen CR Stange KC Nutting PA Competing demands of primary care: a model for the delivery of clinical preventive services J Fam Pract 1994 38 166 171 8308509

[B31] Gillam S Abbott S Banks-Smith J Primary care groups: Can primary care groups and trusts improve health? BMJ 2001 323 89 92 10.1136/bmj.323.7304.89 11451785 PMC1120754

[B32] McKinlay E Plumridge L McBain L McLeod D Pullon S Brown S "What sort of health promotion are you talking about?": a discourse analysis of the talk of general practitioners Soc Sci Med 2005 60 1099 1106 10.1016/j.socscimed.2004.06.041 15589677

[B33] Jacobsen ET Rasmussen SR Christensen M Engberg M Lauritzen T Perspectives on lifestyle intervention: the views of general practitioners who have taken part in a health promotion study Scand J Public Health 2005 33 4 10 10.1080/14034940410028181 15764235

[B34] Coleman T Wynn AT Stevenson K Cheater F Qualitative study of pilot payment aimed at increasing general practitioners' antismoking advice to smokers BMJ 2001 323 432 435 10.1136/bmj.323.7310.432 11520844 PMC37556

[B35] Anderson R New MRC guidance on evaluating complex interventions BMJ 2008 337 944 945 10.1136/bmj.a944 18945728

[B36] Craig P Dieppe P Macintyre S Michie S Nazareth I Petticrew M Developing and evaluating complex interventions: the new Medical Research Council guidance BMJ 2008 337 979 983 10.1136/bmj.a1655 PMC2769032 18824488

[B37] Shiell A Hawe P Gold L Complex interventions or complex systems? Implications for health economic evaluation BMJ 2008 336 1281 1283 10.1136/bmj.39569.510521.AD 18535071 PMC2413333

[B38] Durie R Wyatt K New communities, new relations: the impact of community organization on health outcomes Soc Sci Med 2007 65 1928 1941 10.1016/j.socscimed.2007.05.039 17614171

[B39] McNeill LH Kreuter MW Subramanian SV Social environment and physical activity: a review of concepts and evidence Soc Sci Med 2006 63 1011 1022 10.1016/j.socscimed.2006.03.012 16650513

[B40] Wilson T Holt T Greenhalgh T Complexity science: complexity and clinical care BMJ 2001 323 685 688 10.1136/bmj.323.7314.685 11566836 PMC1121241

[B41] Kroke A Boeing H Rossnagel K Willich SN History of the concept of 'levels of evidence' and their current status in relation to primary prevention through lifestyle interventions Public Health Nutr 2004 7 279 284 10.1079/PHN2003535 15003135

[B42] Beich A Gannik D Malterud K Screening and brief intervention for excessive alcohol use: qualitative interview study of the experiences of general practitioners BMJ 2002 325 870 10.1136/bmj.325.7369.870 12386040 PMC129636

[B43] Wormald H Waters H Sleap M Ingle L Participants' perceptions of a lifestyle approach to promoting physical activity: targeting deprived communities in Kingston-upon-Hull BMC Public Health 2006 6 202 10.1186/1471-2458-6-202 16889657 PMC1560127

[B44] Etz RS Cohen DJ Woolf SH Holtrop JS Donahue KE Isaacson NF Bridging primary care practices and communities to promote healthy behaviors Am J Prev Med 2008 35 S390 S397 10.1016/j.amepre.2008.08.008 18929986

[B45] Cifuentes M Fernald DH Green LA Niebauer LJ Crabtree BF Stange KC Prescription for health: changing primary care practice to foster healthy behaviors Ann Fam Med 2005 3 Suppl 2 S4 11 10.1370/afm.378 16049083 PMC1466978

[B46] Rogers S Humphrey C Nazareth I Lister S Tomlin Z Haines A Designing trials of interventions to change professional practice in primary care: lessons from an exploratory study of two change strategies BMJ 2000 320 1580 1583 10.1136/bmj.320.7249.1580 10845968 PMC27404

[B47] Pronk NP Peek CJ Goldstein MG Addressing multiple behavioral risk factors in primary care. A synthesis of current knowledge and stakeholder dialogue sessions Am J Prev Med 2004 27 4 17 10.1016/j.amepre.2004.04.024 15275669

